# FastPCA: An R package for fast singular value decomposition

**DOI:** 10.21105/joss.09706

**Published:** 2026-03-07

**Authors:** Kimberly R. Ward, Mitchell Hayes, Lauren C Peres, Brooke L Fridley, Steven Eschrich, Alex C Soupir

**Affiliations:** 1 Department of Cutaneous Oncology, Moffitt Cancer Center; 2 Department of Genitourinary Oncology, Moffitt Cancer Center; 3 Department of Cancer Epidemiology, Moffitt Cancer Center; 4 Health Services & Outcomes Research, Children’s Mercy; 5 Department of Pediatrics, University of Missouri-Kansas City School of Medicine; 6 Department of Bioinformatics and Biostatistics, Moffitt Cancer Center

## Summary

The FastPCA package provides an interface to optimized matrix multiplication libraries (libtorch) for the purpose of singular value decomposition. Using FastPCA to perform randomized singular value decomposition (SVD, (Halko, Martinsson, & Tropp, 2011)) with the torch or pytorch backend drastically reduces the computational time compared to base R prcomp and other truncated singular value decomposition. Developed for biological data such as single-cell RNA-sequencing, spatial transcriptomics, or matrix-assisted laser desorption/ionization (MALDI imaging), FastPCA can efficiently and accurately identify the leading singular values in high-dimensional data.

## Statement of Need

Over the last decade, advances in single-cell and spatial profiling technologies have dramatically increased the scale and resolution of biological data (Lim, Wang, Buzdin, & Li, 2025). Multiple custom and commercially available technologies produce tens of thousands of samples (e.g., spots, cells, pixels) and thousands of measured features (i.e., genes, peaks, etc) per sample which can require tens to hundreds of gigabytes (Zaima, Hayasaka, Goto-Inoue, & Setou, 2010). Creating linear or non-linear combinations of the input space in lower-dimensional space enables more efficient downstream analysis. However, this is typically done with packages like irlba (Baglama, Reichel, & Lewis, 2011) where the process of identifying singular values is iterative and single-thread limited. Other packages can improve efficiency by using an API for R (such as reticulate for Python) but is not multithreaded without modification to the source code (Li, Meisner, & Albrechtsen, 2023). The qrpca R package (S. de Souza, Quanfeng, Shen, Peng, & Mu, 2022) uses torch (Falbel & Luraschi, 2020; Paszke et al., 2019) to perform QR-based principal component analysis (PCA), but doesn’t produce truncated singular values resulting in incredibly large memory requirements for large matrices. For biological studies, parameter tuning and rapid iteration (e.g., normalization methods, subclustering for identifying specific cell types) are essential to identify biologically meaningful signals, which typically reside in early dimensions, enabling the avoidance of the full decomposition.

The FastPCA R package was developed to address this critical need. FastPCA has access to the torch backend through R, as well as pytorch with reticulate (Ushey, Allaire, & Tang, 2017). We’ve also included vignettes to demonstrate FastPCA’s utility and functionality (https://acsoupir.github.io/FastPCA/).

## Functionality

FastPCA uses irlba as the default backend for calculating singular values for compatability, but offers access to libtorch via Python pytorch or R torch packages. Using the pytorch or torch backends support setting the number of threads where the backend provides it. There are two functions within FastPCA that aid in the setup of the environments:

- setup_py_env(): creates a Python environment (either with ‘conda’ or ‘virtualenv’) for using in the event that pytorch is wanted for the backend
- start_FastPCA_env(): starts the environment created with setup_py_env() to use for pytorch backend

Then, there are processing functions that work on input matrices or intermediate lists produced by FastPCA:

- prep_matrix(): converts expected data types (rows/columns) into FastPCA format (rows as samples, columns as features). Also supports transformations such as log2 and scaling.
- FastPCA(): performs either exact SVD (though not recommended other than benchmarking) or truncated singular value decomposition with irlba or randomized SVD. Returns a list containing matrices of singular vectors *U* and *V*^*T*^, and vector of singular values *S*
- get_pc_scores(): calculates the principal component scores from the output of FastPCA() (*U* matrix which contains left singular vectors, *S* vector containing the singular values, and *V*^*T*^ matrix of the right singular vectors) which are typically expected for downstream analyses
- umap(): uses either uwot (R) or umap-learn (python) for visualization of the principal component scores

## Research Impact

To demonstrate the improvements of FastPCA, we use our previous data set of single-cell spatial transcriptomics of kidney cancer. The dataset is publicly available on Zenodo and can be downloaded locally to be used (Soupir et al., 2024). Since the main benefits of FastPCA are in its application of singular value decomposition, that is what will be focused on. The counts for each cell were extracted from the Seurat object in the Nanostring assay, which contains the expression of 978 probes (959 genes, 19 negative control probes) in 199,112 cells. Normalization was performed using prep_matrix(), applying a log transformation, scaling (mean centering and unit variance), and transposing for samples to be rows and columns to be the gene features. The full script can be found https://github.com/ACSoupir/FastPCA/blob/main/docs_acs/paper/archive/benchmarking_script.R.

**Figure 1:**
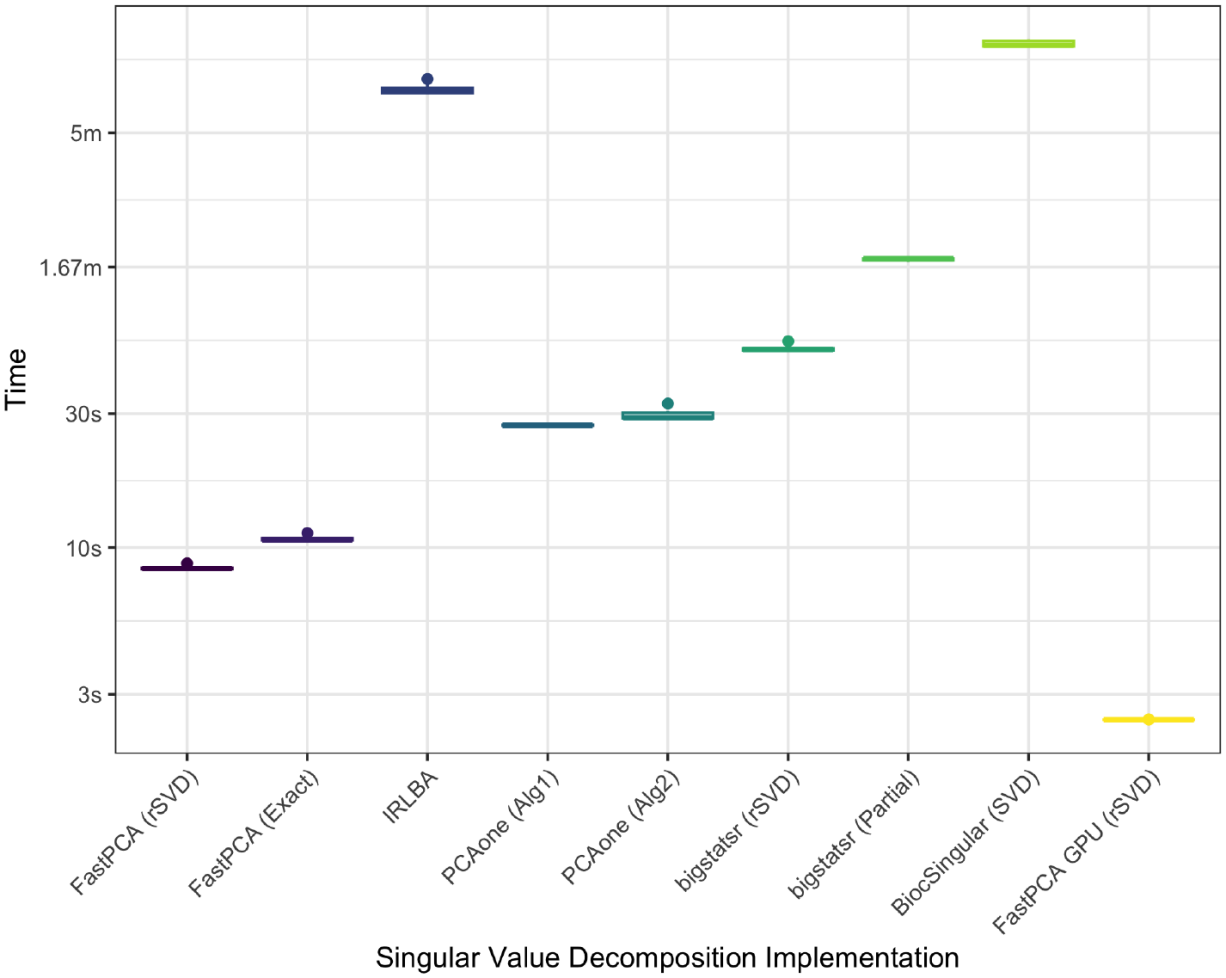
Time of singular value decomposition implementations over 10 replicates.

Experiments were performed using FastPCA, both randomized SVD and exact, irlba, pcaone (Li et al., 2023), and bigstatsr (Privé, Aschard, Ziyatdinov, & Blum, 2018), both partial and randomized partial SVD. Performance measures were collected from a M3 Pro MacBook Pro with 36GB unified RAM. FastPCA (4 cores), bigstatsr partial (1 core) and random (4 cores), irlba (1 core), and pcaone (1 core) were assessed. Time was profiled with bench::mark() function over 10 repetitions for each implementation. The elapsed time of FastPCA calculating the singular value decomposition on the full data took 10.74 seconds on average (range 10.61 to 11.27 seconds) and the randomized SVD with FastPCA taking 8.46 (8.35 to 8.79) seconds on average Table 1, Figure 1). The next fastest were the implementations with PCAone which took 27.29 (27.1 to 27.54) and 29.58 (28.59 to 32.59) seconds on average for “Alg1” and “Alg2”, respectively.

The pytorch backend also has the ability to use a CUDA-enabled GPU (using device = “GPU”), leveraging the highly parallel processing commonly used in deep learning applications (Paszke et al., 2019). Enabling GPU acceleration with FastPCA further improves compute time to 2.44 seconds. While current GPU implementation significantly improves speed for same operation (CPU time between 8.35 and 8.27 seconds; GPU time of 2.44 seconds), it is limited to CUDA due to lack of access to other hardware. Future development of FastPCA aims to be hardware agnostic through added support or adding other hardware agnostic backends like tinygrad (https://tinygrad.org/).

Reconstruction error was calculated for all implementations. Because the three matrices (left eigenvectors, eigenvalues, and right eigenvectors) should contain all of the information decomposed from the original matrix when calculated in full, exact FastPCA was used as reference. To determine how much information was captured in the truncated methods compared to the full decomposition, the output from exact FastPCA was directly truncated to the first 100 dimensions and sum of squares error ratio was calculated (reduced SSE / exact truncated SSE; Table 2; (Eckart & Young, 1936)). Values of 1 are interpreted as containing the same variance in the reconstructed data as the first 100 dimensions of the truncated exact SVD, and values greater than 1 indicate how much error over optimal reconstruction the method or implementation had. These demonstrate that irlba with it’s iterative approach captures the first 100 dimensions extremely well (1.000) compared to the exact SVD; similar with the bigstatsr implementations. FastPCA randomized SVD and PCAone approaches contain slightly greater error (~0.14% to ~0.33% greater than the optimal reconstruction with exact FastPCA) just like BiocSingular randomized SVD (~0.33%). These errors indicate that with FastPCA being more than 50x faster using the randomized SVD approach than IRLBA, it is within the error of other common implementations, and while being greater than 40x faster using the exact approach perfectly reconstructs the original data.

Further, for the first 100 singular values, and first and second left singular vectors (samples), we used the concordance correlation coefficient (CCC) to demonstrate agreement of each with the exact FastPCA. Singular values produced by irlba and both implementations from bigstatsr were identical to those from the full SVD (Table 3). Again, FastPCA using randomized SVD, both algorithms from pcaone, and BiocSingular deviated slightly from the exact yet were considered “almost perfect” having CCC values greater than 0.99 (actually > 0.9999; (Akoglu, 2018)). For both principal component (PC) 1 and 2, the CCC was 1.0000 indicating perfect agreement (PCs calculated by multiplying the left singular vectors by the singular values).

To demonstrate that the FastPCA exact is mathematically sound for the comparison above, we assessed it’s error compared to the original expression matrix. The absolute error (using the r norm function with type = “F”) of the reconstructed data from the exact method is 2.11e-09. Relative error, calculated by dividing absolute error by square root of variance within the original data, was calculated to be 1.51e-13.

## Conclusion

FastPCA provides an accelerated route to principal component analysis on large matrices by coupling randomized SVD with torch/pytorch backends and a familiar R API. In our kidney cancer spatial-transcriptomics benchmark (199,112 cells × 978 features), FastPCA’s randomized SVD achieved 8.5 s mean runtime on commodity hardware while matching the early singular values from exact decompositions and widely used methods (IRLBA in irlba). Memory use remained modest for FastPCA’s randomized SVD implementation (321.4 MB), with predictable increase for exact decompositions when full matrices are returned. These results, together with setup helpers (optional Python via reticulate, otherwise R-only), make FastPCA a practical default for exploratory analyses and iterative pipelines (e.g., normalization alternatives, sub-clustering, visualization) where the top components contain the most biological information. Future work will extend functionality to sparse-matrix coverage and GPU pathways.

## Figures and Tables

**Table 1:** Time to calculate singular value decomposition using different R packages and implementations over 10 replicates.

Implementation	Min	Median	Mean	Max
FastPCA (rSVD)	8.35s	8.43s	8.46s	8.79s
FastPCA (Exact)	10.61s	10.65s	10.74s	11.27s
irlba	413.15s	423.73s	429.99s	466.80s
pcaone (Alg1)	27.1s	27.27s	27.29s	27.54s
pcaone (Alg2)	28.59s	29.01s	29.58s	32.59s
bigstatsr (rSVD)	50.0s	50.8s	51.15s	54.34s
bigstatsr (Partial)	104.08s	106.94s	106.43s	107.73s
BiocSingular (rSVD)	606.71s	615.53s	623.30s	646.58s
FastPCA GPU (rSVD)	2.44s	2.44s	2.44s	2.44s

**Table 2:** Reconstruction error of the truncated singular value decomposition. FastPCA using the exact approach was used as reference as it produces the full singular value decomposition.

Implementation	SSE Ratio
FastPCA (rSVD)	1.003191
FastPCA (Exact)	–
irlba	1.000000
pcaone (Alg1)	1.003316
pcaone (Alg2)	1.001383
bigstatsr (rSVD)	1.000000
bigstatsr (Partial)	1.000000
BiocSingular (rSVD)	1.003275

**Table 3:** Concordance correlation coefficient estimates for how well each implementation agrees with the true full FastPCA singular value decomposition on the singular values and principal component 1 and 2 values.

Implementation	Singular Values	Principal Component 1	Principal Component 2
FastPCA (rSVD)	0.9999	1.0000	1.0000
FastPCA (Exact)	–	–	–
irlba	1.0000	1.0000	1.0000
pcaone (Alg1)	0.9999	1.0000	1.0000
pcaone (Alg2)	1.0000	1.0000	1.0000
bigstatsr (rSVD)	1.0000	1.0000	1.0000
bigstatsr (Partial)	1.0000	1.0000	1.0000
BiocSingular (rSVD)	0.9999	1.0000	1.0000

## Data Availability

Review
Repository
Archive
